# Supplementary figures and images for: Serum Androgen Metabolites Correlate with Clinical Variables in African and European American Men with Localized, Therapy Naïve Prostate Cancer
Source: Metabolites. 2023 Feb 16;13(2):284. doi: 10.3390/metabo13020284 (PMC9962438; doi:10.3390/metabo13020284)

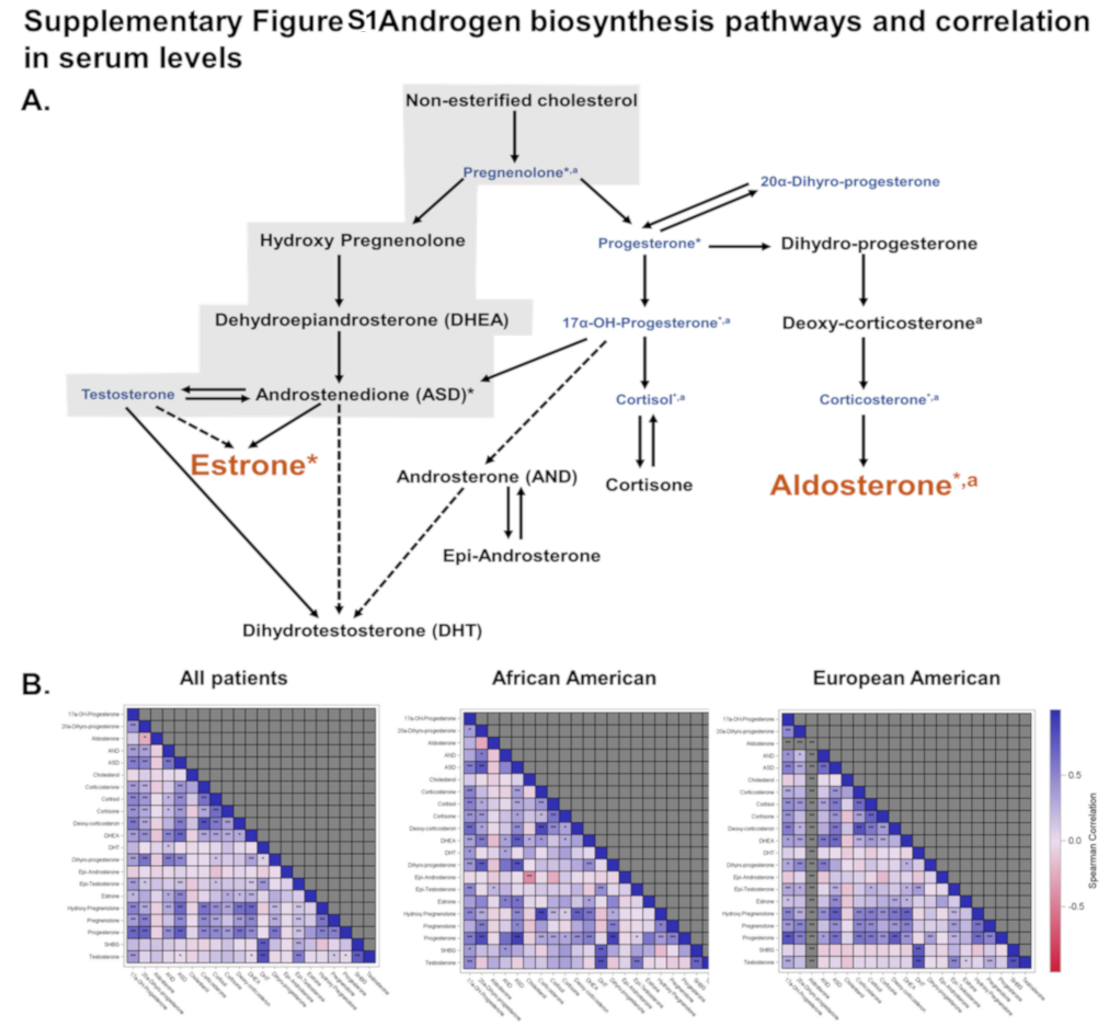

Supplement: Supplementary file 1 [file metabolites-13-00284-s001.zip › Figure S1.tif]

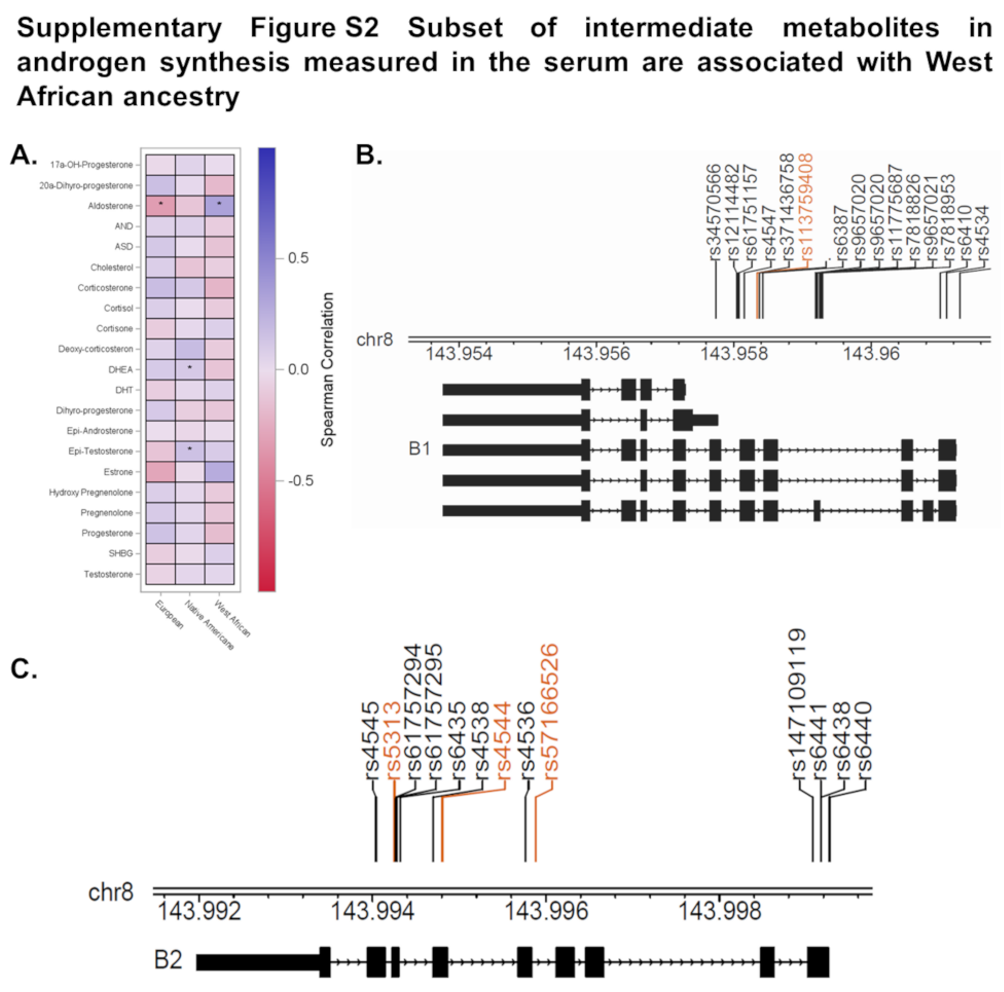

Supplement: Supplementary file 1 [file metabolites-13-00284-s001.zip › Figure S2.tif]

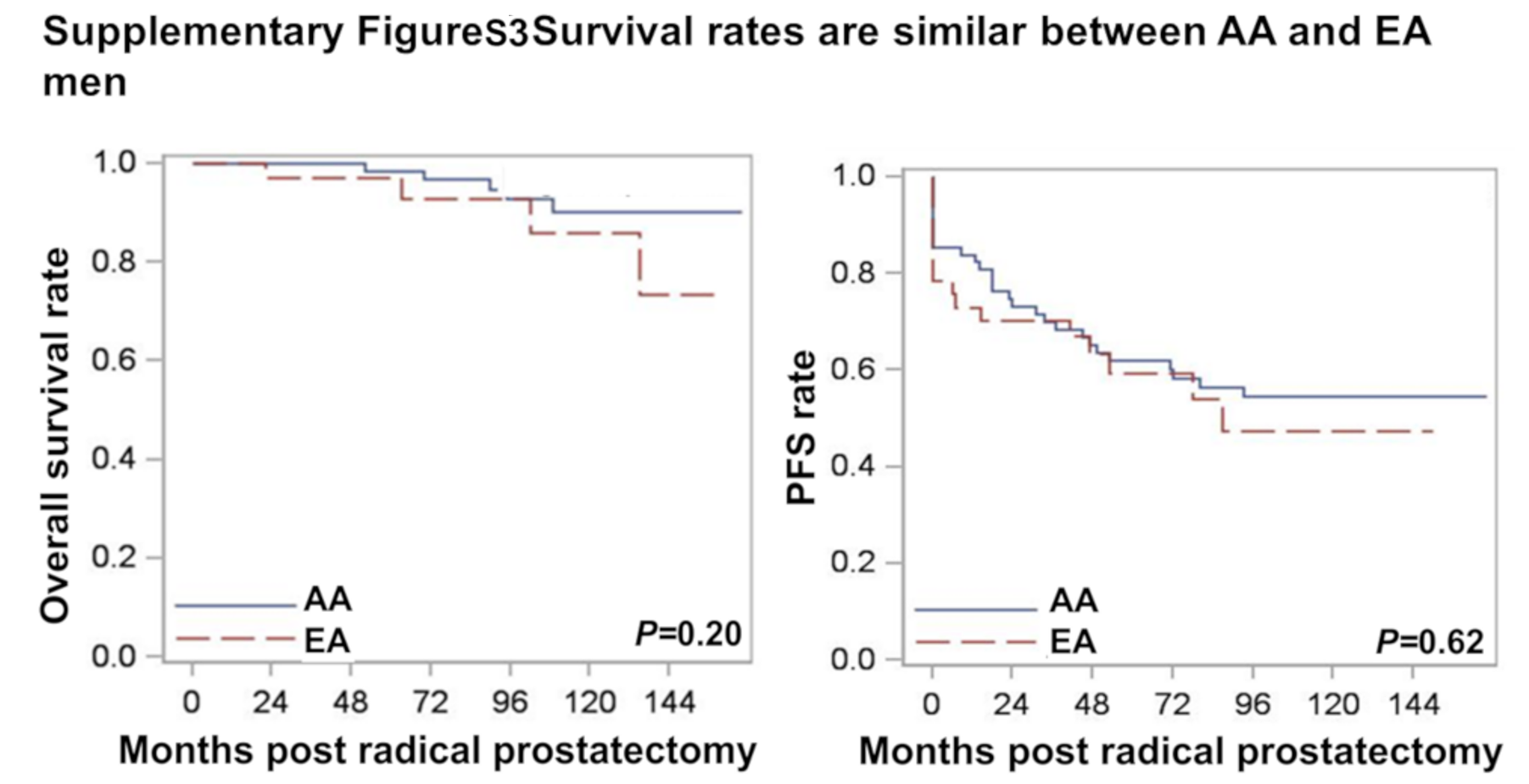

Supplement: Supplementary file 1 [file metabolites-13-00284-s001.zip › Figure S3.tif]
